# Coordinated performance measurement and improvement efforts in California's safety net systems: early experience and lessons

**DOI:** 10.1186/1748-5908-10-S1-A59

**Published:** 2015-08-20

**Authors:** Urmimala Sarkar, David Lown, Sara Ackerman, Courtney Lyles, Gato Gourley, Dean Schillinger, Margaret A Handley

**Affiliations:** 1 Center for Vulnerable Populations, Division of General Internal Medicine, University of California San Francisco, San Francisco, CA 94110, USA; 2 The California Health Care Safety Net Institute, Oakland, CA 94607, USA; 3 Department of Social and Behavioral Sciences, School of Nursing, University of California San Francisco, San Francisco, CA 94143, USA; 4 Department of Epidemiology and Biostatistics, University of California San Francisco, San Francisco, CA 94158, USA

## Introduction

Safety-net health systems often lack incentives and resources to support performance measurement and improvement activities. The California Delivery System Reform Incentive Program (DSRIP) is a pay-for-performance initiative that incentivizes the state's public health care systems to improve quality of care. Health system participants must report on metrics in both inpatient and outpatient settings. To enhance DSRIP participants' capacity to engage in (1) best practices to improve quality of care and (2) reporting of DRSIP-required metrics, the California Association of Public Hospitals and UCSF established the Public Healthcare systems Evidence Network and Innovation eXchange (PHoENIX), with funding from AHRQ. PHoENIX priorities are meeting the HEDIS Medicare PPO 90^th ^percentile thresholds for mammography screening (76.6%) and cholesterol control (LDL-C) for diabetes (<100 mg/dl, 62.2%).

## Methods

Using a mixed methods approach and applying the Consolidated Framework for Implementation Research to characterize improvement efforts, we identified barriers and enablers for meeting the performance metrics.

## Results

Engagement in improvement was high, with all systems implementing quarterly performance measurement and participating in web-based and in-person learning sessions. Barriers include: lack of personnel for data reporting/management; structural barriers, such as fragmented electronic health records, lack of equipment (e.g., mammogram machines); competing priorities, such as providing access to care for the newly insured; and a change in the national guideline for cholesterol control. Enablers included: strong leadership, integrated data systems. To date, 82% of the health systems improved their performance on mammography and 53% improved on LDL.

## Conclusion

Public health systems can collectively engage in performance measurement and improvement; implementation science methods identify barriers and enablers of change which can be applied to real-world improvement efforts.

